# Monocyte Chemotactic Protein 1 in Plasma from Soluble *Leishmania* Antigen-Stimulated Whole Blood as a Potential Biomarker of the Cellular Immune Response to *Leishmania infantum*

**DOI:** 10.3389/fimmu.2017.01208

**Published:** 2017-09-29

**Authors:** Ana V. Ibarra-Meneses, Carmen Sanchez, Jorge Alvar, Javier Moreno, Eugenia Carrillo

**Affiliations:** ^1^WHO Collaborating Centre for Leishmaniasis, Centro Nacional de Microbiología, Instituto de Salud Carlos III, Madrid, Spain; ^2^Drugs for Neglected Diseases Initiative (DNDi), Geneva, Switzerland

**Keywords:** chemokines, cytokines, biomarker, leishmaniasis, whole blood assay, asymptomatic infection, treatment, monocyte chemotactic protein 1

## Abstract

New biomarkers are needed to identify asymptomatic *Leishmania* infection as well as immunity following vaccination or treatment. With the aim of finding a robust biomarker to assess an effective cellular immune response, monocyte chemotactic protein 1 (MCP-1) was examined in plasma from soluble *Leishmania* antigen (SLA)-stimulated whole blood collected from subjects living in a *Leishmania infantum*-endemic area. MCP-1, expressed 110 times more strongly than IL-2, identified 87.5% of asymptomatic subjects and verified some asymptomatic subjects close to the cutoff. MCP-1 was also significantly elevated in all patients cured of visceral leishmaniasis (VL), unlike IL-2, indicating the specific memory response generated against *Leishmania*. These results show MCP-1 to be a robust candidate biomarker of immunity that could be used as a marker of cure and to both select and follow the population in vaccine phase I–III human clinical trials with developed rapid, easy-to-use field tools.

## Introduction

### New Biomarkers Capable of Identifying Specific Cellular Immunity to *Leishmania* in the Field are Necessary

Visceral leishmaniasis (VL) is the most severe form of a spectrum of neglected tropical diseases caused by protozoan intracellular parasites of the genus *Leishmania*. The adaptive immune response to *Leishmania* infection is associated with the activity of T lymphocytes that produce cytokines or chemokines that activate or dampen the antiparasitic activity of macrophages, the primary resident cell for *Leishmania* (1). In addition, T lymphocytes are critical for cure and generation of a protective immune response (2). Despite the numerous studies on the cellular immunity to *Leishmania* infection, very few results have been carried out to development of useful field test for the assessment of such protective immune response. In this context, biomarkers able to reveal the outcome of leishmaniasis treatment are also urgently needed. Not only would they be useful when making clinical decisions regarding the continuation of treatment, they would allow trials of new drugs or treatment regimens to be shortened, since the expression of this immunity biomarkers could confirm that recovery is completed and no relapsed should be expected. At the moment, no vaccines exist for human VL, although different attempts are in progress (3). In parallel to vaccine development, it is also important to establish those tools needed to assess the adaptive immunity elicited by the vaccine candidate as well as to screen in advance the population already exposed and naturally immunized against the parasite. This would presumably avoid potential bias of phase III clinical trials. Furthermore, in endemic areas, most of the infected population is asymptomatic, and the identification and management of such subjects has become an increasingly important challenge in VL control.

Whole blood immunoassays (WBA) provide a non-invasive, non-sensitizing test for assessing T cell-mediated immunity against *Leishmania* that is easily translatable to the field. Levels of IFN-γ and IL-2 in the plasma of soluble *Leishmania* antigen (SLA)-stimulated whole blood have been identified as biomarkers for use in WBA, allowing the detection of asymptomatic subjects in areas where *Leishmania* species likely to cause VL are endemic (4, 5). Recently, the production of IFN-γ in plasma from SLA-stimulated whole blood has also been described to gradually but significantly increase after the successful treatment of patients infected with *L. donovani* (2) and *L. infantum* (3). However, both IFN-γ and IL-2 are cytokines produced in small amounts in these WBA assays, and more abundant and robust biomarkers for measuring immunity to *L. infantum* would be preferable.

Chemokines are produced early in the responses to *Leishmania* parasites, providing a signal for the initiation of the immune response (6). The chemokine CC motif ligand 2 (CCL2), also known as monocyte chemotactic protein 1 (MCP-1), plays a variety of roles in host defense against *Leishmania* (7). Recently, plasma MCP-1 concentrations before and after effective treatment against *L. donovani* have been described as similar (8), but the biomarker potential of chemokines produced in SLA-stimulated whole blood has not been explored in leishmaniasis. Certainly, MCP-1 has been proposed as a biomarker for monitoring immunity to *M. tuberculosis* after whole blood stimulation (9). In addition, this chemokine is expressed at levels many times higher than IFN-γ or IL-2, potentially making it a more sensitive biomarker.

Taking into account the high and specific expression of MCP-1 by *Leishmania*-stimulated T-cells, we have investigated the use of this chemokine as a specific, easily detected biomarker of the cellular immune response against *L. infantum* infection useful to identify asymptomatic subjects living in an endemic area, and as a means of monitoring the specific immune response following treatment for VL. Such biomarkers could be an irreplaceable tool for the screening of previously immunized/non-immunized individuals during phase III clinical trial of vaccines as well in new drugs clinical trials.

## Materials and Methods

### Study Group

Blood samples from asymptomatic subjects (*N* = 40) and negative controls (*N* = 20) were collected in 2016 at the blood bank of *Hospital de Fuenlabrada*, Spain. The criteria for distinguishing asymptomatic subjects from those not infected was a positive “*in vitro* peripheral blood mononuclear cell proliferation assay” (CPA), as previously described (4). Blood was also taken from seven subjects with active leishmaniasis before and at 6 months after starting treatment with amphotericin B (10). Other seven samples from CVL subjects were included for comparison with AS and NVL. In these patients, blood plasma was isolated by standard centrifugation at then collected and stored at −80°C. Conventional techniques for diagnosis of VL were performed as described previously (11).

### Whole Blood Stimulation Assay

Monocyte chemotactic protein 1 and IL-2 were sought in the plasma of SLA-stimulated whole blood taken from the asymptomatic patients and controls, as well as in the natural plasma and the plasma of SLA-stimulated whole blood taken from the patients with VL before and after cure. For SLA stimulation, 500 µL of whole blood was placed in a tube containing 10 µg/mL SLA, as previously described (4); a similar tube was prepared without SLA as a negative control. After incubation at 37°C for 24 h, the plasma was collected, diluted 1:10 PBS with 3% BSA.

### Cytometric Quantification

Monocyte chemotactic protein 1 and IL-2 were quantified in 50 µL of natural plasma and SLA-stimulated plasma by using the BD Cytometric Bead Array Human Flex Set (Becton Dickinson Biosciences, USA) following the manufacturer’s instructions. The results for whole blood assay were expressed as the difference between the SLA-stimulated and negative control subject plasma for MCP-1 and IL-2 concentrations.

### Statistical Analyses

The ability of MCP-1 to identify asymptomatic subjects was determined by calculating the area under the receiver operating characteristic (ROC) curve (AUC) and the 95% confidence interval (95% CI).

The MCP-1 concentrations in the natural plasma and plasma from SLA-stimulated whole blood taken from the seven VL patients were determined at the time of diagnosis of VL and after VL cure (defined as no relapse within 6 months after beginning treatment), and compared using the Wilcoxon test. We compared the MCP-1 and IL-2 concentrations in VL, CVL, asymptomatic, and NVL subjects using the Mann–Whitney *U* test. Significance was set at *p* ≤ 0.05.

## Results

### MCP-1 in SLA-Stimulated Plasma is Increased after Cure, Unlike IL-2

**Figure 1:**
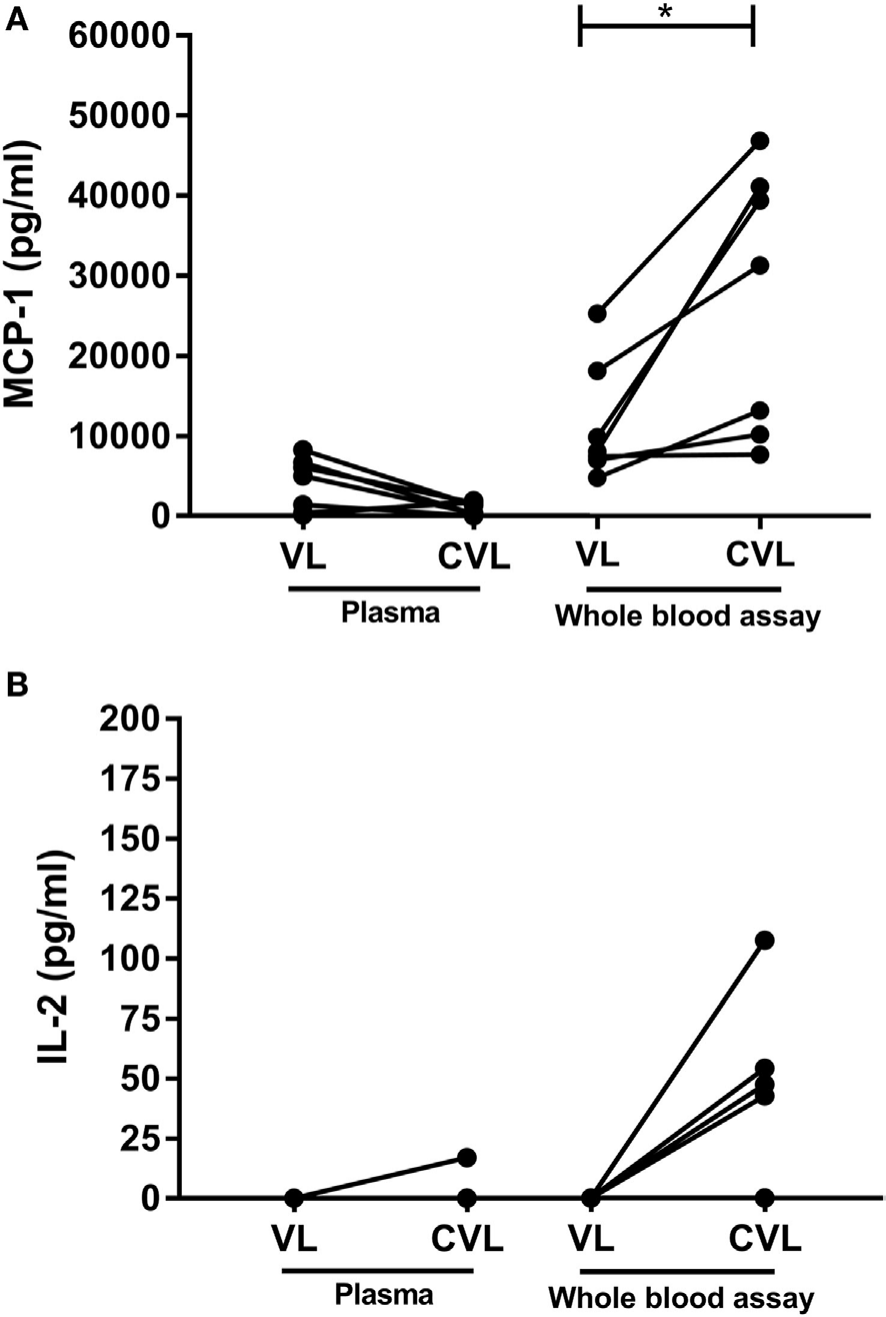
Levels of monocyte chemotactic protein 1 (MCP-1) **(A)** and IL-2 **(B)** in natural plasma and soluble *Leishmania* antigen (SLA)-stimulated plasma in active visceral leishmaniasis (VL) patients compared to cured VL (CVL) after 6 months of treatment. Levels of MCP-1 and IL-2 before and after treatment were analyzed using Wilcoxon matched pairs signed rank test, *p < 0.05.

Plasma circulating levels of MCP-1 were similar before and after treatment of the patients with VL (Figure 1A: median 5,029 pg/mL and 1,368 pg/mL; *p* = 0.1094). No plasma circulating levels of IL-2 were found in most VL and cured VL (CVL) (Figure 1B). However, MCP-1 levels in SLA-stimulated plasma showed a significant increase after VL cure at 6 months in all treated patients (Figure 1A: median 8,396 and 32,498 pg/mL; *p* = 0.0156). Only three out of seven cured patients increased IL-2 levels in SLA-stimulated plasma above the cutoff, while four of them had same levels that in active status (median 0 pg/mL) (Figure 1B).

The AUC for MCP-1 was 0.9011 (95% CI: 0.7676–1.000; *p* = 0.0038), the sensitivity was 76.92% (95% CI 46.19–94.96), and specificity was 85.71% (95% CI 42.13–99.64). IL-2 showed AUC of 0.6786 (95% CI: 0.4601–0.897; *p* = 0.1814), the sensitivity was 43.75% (95% CI 19.75–70.12) and specificity was 100% (95% CI 59.04–1.00).

### MCP-1 is Expressed at Higher Concentrations in CVL and Asymptomatic Subjects Compared to VL Patients and Negative Control Individuals

**Figure 2:**
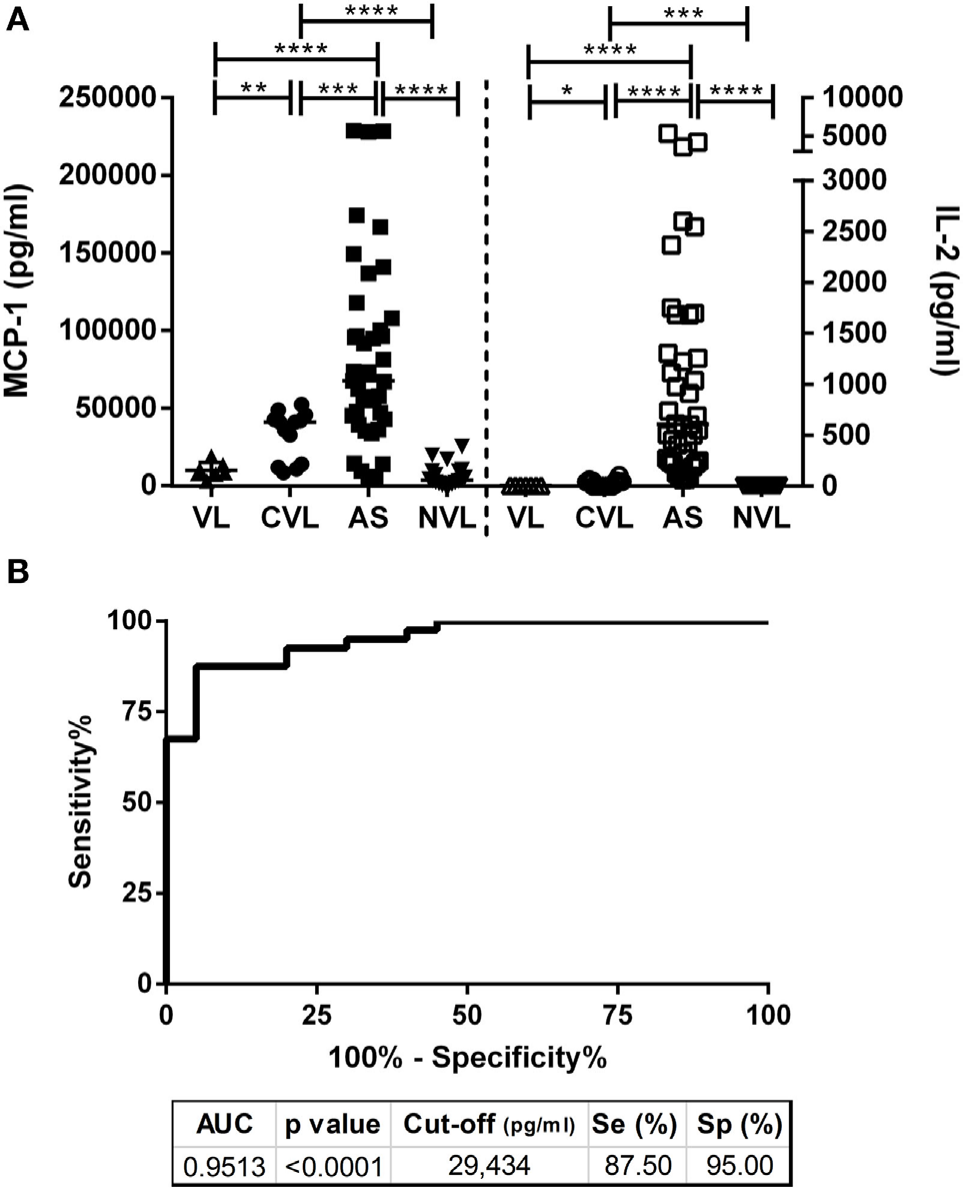
**(A)** Production of MCP-1 and IL-2 in plasma from SLA-stimulated whole blood taken from active visceral leishmaniasis (VL, *n* = 7), cured visceral leishmaniasis (CVL; *n* = 14), asymptomatic participants (AS; *n* = 40) and negative controls (NVL; *n* = 20). Horizontal bars represent median concentrations. Each dot represents one subject. Data were analyzed using the Mann–Whitney *U* test. *p < 0.05; **p < 0.01; ***p < 0.001; ****p < 0.0001. **(B)** Receiver operating characteristic (ROC) curve showing the sensitivity and specificity of monocyte chemotactic protein 1 (MCP-1) in the detection of asymptomatic individuals. The AUC of MCP-1, *p*-value, cut-off, sensitivity, and specificity are indicated on the table.

The stimulation of whole blood with SLA led to significantly higher plasma MCP-1 concentrations in cured VL and asymptomatic individuals compared to the active VL and negative controls (median 40,863 and 67,386 vs. 8,396 and 3,442 pg/mL, respectively). Compared to IL-2 levels (median 0 pg/mL for VL and CVL; 608.6 pg/mL for asymptomatic individuals, and 0 pg/mL for negative controls; *p* < 0.0001), the median values of MCP-1 in plasma were at least 110 times higher than IL-2 for asymptomatic subjects (Figure 2A).

In the detection of asymptomatic individuals, the analysis of MCP-1 levels returned an AUC of 0.9513 (95% CI 0.9022–1.0000; *p* < 0.0001), its sensitivity was 87.50% (95% CI 73.20–95.81), and its specificity 95% (95% CI 75.13–99.87). The calculated cutoff (29,434 pg/mL) for the MCP-1 concentration in plasma from SLA-stimulated whole blood identified 87.5% (35/40) of the asymptomatic individuals (Figure 2B), while IL-2 showed 100% sensitivity and specificity (4). Despite the difference in sensitivity, 4 out of 40, asymptomatic subjects (10%) had IL-2 levels close to the cutoff (cutoff = 50.31 pg/mL; data: 66.25; 68.17; 96.71; 98.77 pg/mL) while, on the contrary, showing a high MCP-1 (cutoff = 29,434 pg/mL; data: 73,520; 228,781; 42,723; 94,833 pg/mL).

## Discussion

### MCP-1: A Specific and Highly Expressed Biomarker after *Leishmania* Stimulation

New biomarkers capable of identifying asymptomatic *L. infantum-*infected individuals are necessary for the development of sensitive and rapid tools for use in the field. One of the best to date is the quantification of IL-2 in plasma from SLA-stimulated whole blood assays. Although IL-2 is 100% sensitive and specific (4), the concentration of this biomarker is low, and a more robust biomarker produced in much larger quantities would be preferable. The present results show that the concentration of MCP-1 in plasma from SLA-stimulated whole blood can identify asymptomatic individuals and is expressed much more strongly that IL-2. Although the sensitivity is 87.5%, MCP-1 showed high levels in those asymptomatic subjects with values of IL-2 near of the cutoff and it could be useful to further verify their status in a precise moment or along their follow-up in a *Leishmania* endemic area.

Given its capacity to activate anti-*Leishmania* macrophage killing mechanisms, MCP-1 has been widely studied as a biomarker of cure in cutaneous leishmaniasis. High MCP-1 expression in the skin is described in localized, self-healing cutaneous lesions (12), but no detectable MCP-1 is found in diffuse, non-healing cutaneous lesions (13, 14). In VL, however, pretreatment and posttreatment plasma levels of MCP-1 have been reported similar (15). We have, therefore, examined the concentration of MCP-1 in natural plasma and plasma from SLA-stimulated whole blood assays, and the results for the latter showed MCP-1 levels to be significantly increased after VL cure. This is a clear advantage regarding the measure of IL-2 and promotes MCP-1 from SLA-stimulated whole blood assays as a potential biomarker to be explored for the follow-up of the treatment until cure. Although IFN-γ in plasma from SLA-stimulated blood has recently been described to significantly increase after the successful treatment of patients infected with *L. donovani* (2) and *L. infantum* (3), the patent difference of levels between IFN-γ and MCP-1 suggested the inclusion of this chemokine in further studies with higher number of patients.

Taking into account all of the above, the combined use of both IL-2 and MCP-1 after whole blood assay could led to get an easy-to-use field test to assess cell immunity to *Leishmania*. This opens up the possibility of developing simpler detection platforms as immunochromatographic rapid tests.

Although immunodeficiency may seriously affect host biomarker expression, IFN-γ and TNF-α in plasma from SLA-stimulated whole blood assay have been found useful for following up solid organ transplant recipients treated for VL (11). However, the literature contains some evidence that MCP-1 could also be used as a biomarker of cure in immunodepressed subjects. In patients with HIV-1 infection, MCP-1 levels and plasma viral load have been shown to diminish after successful indinavir treatment (16). Further investigation into the use of MCP-1 and other chemokines for the monitoring of immunity in immunosuppressed patients with VL is urgently needed.

In conclusion, the combination of both whole blood stimulation assay and the analysis of specific adaptive immunity biomarkers represent an accurate approach to assess *Leishmania*-specific immunity in field work. MCP-1 in this context is a promising, robust biomarker for confirming asymptomatic individuals exposed to *L. infantum*. It may also provide a marker of the efficacy of treatment for VL. The quantities in which MCP-1 is produced might allow new easy-to-use field tools in the control of *Leishmania* and its combined detection with IL-2 will increase the sensitivity of a test of immunity. Further studies are required to validate the combined use of these biomarkers in other endemic areas and to confirm its potential to detect the cellular immune response in immunocompetent and immunosuppressed subjects.

## Ethics Statement

This study was approved by the *Hospital de Fuenlabrada* (Madrid) Ethics and Research Committee (APR 12-65 and APR14-64); all participants gave their written informed consent to be involved.

## Author Contributions

AI-M, JA, JM, and EC designed the study; AI-M and CS performed experiments and analyzed data; AI and EC wrote the manuscript; and AI-M, JA, JM, and EC interpreted the data.

## Conflict of Interest Statement

The authors declare that the research was conducted in the absence of any commercial or financial relationships that could be construed as a potential conflict of interest.
