# Supplementary material for: Monocyte Chemotactic Protein 1 in Plasma from Soluble Leishmania Antigen-Stimulated Whole Blood as a Potential Biomarker of the Cellular Immune Response to Leishmania infantum
Source: Front Immunol. 2017 Sep 29;8:1208. doi: 10.3389/fimmu.2017.01208 (PMC5626820; doi:10.3389/fimmu.2017.01208)
Supplement: Supplementary file 1 [file table_1.docx]

**Supplementary Table 1. Clinical characteristics of the study population.**

| **Group** | **VL** | **CVL** | **AS** | **NVL** |
| --- | --- | --- | --- | --- |
| **No. of subjects** | 7 | 14 | 40 | 20 |
| **Subjects age (y) (median/SD)** | 44 ± 10 | 45 ± 10 | 41 ± 12 | 40 ± 14 |
| **No. (%) of CPA (+)** | 0 | 100 | 100 | 0 |
| **No. (%) of qPCR (+)** | 100 | 0 | 0 | 0 |
| **No. (%) of rK39 (+)** | 62 | 43 | 0 | 0 |
| **No. (%) of IFAT (+)** | 69 | 21 | 0 | 0 |

VL, visceral leishmaniasis;

CVL, cured visceral leishmaniasis;

AS, asymptomatic individuals;

NVL, negative control subjects;

CPA, 'in vitro peripheral blood mononuclear cell proliferation assay'

qPCR, quantitative PCR for the small subunit rRNA gene of *Leishmania*;

rK39, rK39 dipstick (InBIOS International, Seattle, WA);

IFAT, *Leishmania* indirect fluorescent antibody test.
